# Terminal differentiation into adipocyte and growth inhibition by PPARγ activation in human A549 lung adenocarcinoma cells

**DOI:** 10.1080/19768354.2020.1847731

**Published:** 2020-12-02

**Authors:** Dae-Young Kim, Sun-Ha Moon, Jang-Ho Han, Mi-Jeong Kim, Seong-Ju Oh, Dinesh Bharti, Sung-Ho Lee, Jong-Kuen Park, Gyu-Jin Rho, Byeong-Gyun Jeon

**Affiliations:** a Department of Biology Education, Gyeongsang National University, Jinju, Republic of Korea; b OBS/Theriogenology and Biotechnology, Gyeongsang National University, Jinju, Republic of Korea; c Division of Life Science, Gyeongsang National University, Jinju, Republic of Korea; d Department of Chemistry Education, Gyeongsang National University, Jinju, Republic of Korea; e Institute of Education, Gyeongsang National University, Jinju, Republic of Korea

**Keywords:** Human, A549 cells, adipocyte, differentiation, cell growth

## Abstract

The present study investigated the terminal differentiation capacity into adipocytes and subsequent growth inhibition in A549 cancer cells treated with pioglitazone (PGZ), a PPARγ activator. The rate of cell growth in A549 cells was significantly (*P *< .05) inhibited in concentrations above 10 μM PGZ while maintaining less cytotoxic effects in MRC-5 fibroblasts. Following 50 μM PGZ treatment, population doubling time (PDT) was significantly (*P* < .05) increased by inhibition of cell growth, as per increasing PGZ exposure time by up to 4 weeks. The adiposome-like vesicles were commonly observed in the PGZ-treated A549 cells, and the vesicles were highly stained with Oil-Red O solution. In addition, the cell size and expression of GLUT4 and PPARγ were significantly (*P* < .05) increased, as per increasing PGZ exposure time by up to 4 weeks. The significant (*P* < .05) down-regulation of telomerase activity and up-regulation of senescence-associated β-galactosidase (SA β-GAL) activity was displayed in the PGZ-treated A549 cells, as per increasing PGZ exposure time by up to 4 weeks. The G1 phase of the cell cycle was also significantly (*P* < .05) increased in the PGZ-treated A549 cells compared with untreated A549 cells. The present results have demonstrated that activation of PPARγ using PGZ induces cellular differentiation into adipocytes and inhibits cell growth in the A549 cancer cells. The terminal differentiation into adipocytes could offer potent chemotherapy in the cancer cells showing high glucose metabolism.

## Introduction

The cancer diseases are globally a leading cause of death and the cancer of various types is continually increased due to several factors, including the increase of body weight, tobacco, alcohol and so on (Bray et al. 2018). Various types of cancer treatments, including surgery, chemotherapy, radiation therapy, and immunotherapy, have been currently tried, depending on the type of cancer. Further combined treatments, such as surgery with radiation therapy and/or chemotherapy have also been applied for growth inhibition of cancer cells. The chemotherapy using chemical drug with cellular cytotoxicity is most powerful and useful cancer treatment. Recently, several chemical drugs stimulating immune system of body have been successfully applied for cancer treatment with less cellular cytotoxicity (Buchbinder and Desai 2016).

Cancer cells may attain immortal status via various types of genetic modifications and disorders from primarily differentiated mortal somatic cells, and immortality is main characterization of cancer cells (Ilango et al. 2020). Immortality in cancer cells is highly related with over-expression of telomerase activity (Saretzki 2018). Thus, the cell growth and division-targeting chemical drugs are widely used for cancer chemotherapy (Schirrmacher 2019). However, chemicals used in chemotherapy treatment usually affects the growth of both the cancerous and normal (non-cancerous) cells while displaying common side effects including nausea, vomiting, diarrhea, hair loss and so on (Huang et al. 2017). Therefore, anti-cancer chemicals need to be developed which can cause low cytotoxicity in the normal cells while effectively destroying targeted cancer cells.

Embryonic stem cells (ESCs) or induced pluripotent stem cells (iPSCs) commonly exhibit self-renewal for unlimited cell growth and differentiation capacity to become various cell types under specific inductions (Bilic and Izpisua Belmonte 2012). However, this peculiar self-renewal and unlimited cell growth capacity gradually disappears when these cells (ESCs and iPSCs) are terminally differentiated into targeted lineages (He et al. 2009). Previous studies demonstrated that the mouse 3T3-L1 pro-adipocytes with unlimited cell growth are easily differentiated to adipocytes and cell growth is arrested in the differentiated 3T3-L1 cells (Reichert and Eick 1999; Kim et al. 2018). Like 3T3-L1 cells, most of the cancer cells acquire increased metabolism activity to support their unlimited high cell growth capacity. For this, cancer cells use glucose as an energy source for aerobic glycolysis pathway, rather than oxidative phosphorylation pathway (Shaw 2006) and they preferentially express various glucose transporters and enzymes for high glucose metabolism pathway (Cho et al. 2013). In accordance with these observations, drugs targeting glucose metabolism and angiogenesis inhibition have been continually developed (Hamanaka and Chandel 2012). Our group previously demonstrated that several cancer cells (especially A549 lung adenocarcinoma) can be easily differentiated to adipocyte-like cells with high glucose uptake and adiposome vesicles when treated with chemicals such as synthetic cortisol, dexamethasone (DEX) while displaying subsequent cell growth arrest (Kim et al. 2018). Accordingly, we assumed that the unlimited cell cancer cell growth can be gradually disappeared during their differentiation into specific cell types, such as mesenchymal adipocytes. The cellular differentiation into adipogenesis is a multiple process which includes involvement of activated signal pathway and transcription factors, and nuclear receptor peroxisome proliferator-activated receptor γ (PPARγ) is a main regulator in adipogenesis process (Fajas et al. 2001). Pioglitazone (PGZ) is an anti-hyperglycemic agent, commonly used for the treatment of type 2 diabetes (DeFronzo et al. 2011). Further, PGZ was reported to facilitate adipogenesis in the 3T3-L1 cells via PPARγ activation, therefore acts as a PPARγ agonist (Lee et al. 2006; Kudoh et al. 2011).

In the present study, A-549 lung adenocarcinoma cells were induced with PPARγ agonist, PGZ, and were further evaluated for morphological alterations and Oil Red O staining with expression of metabolism-related transcripts. Further, induced adipocytes were examined for the fundamental cancerous cell properties, including cell proliferation rate by population doubling time (PDT) and cell migration assay, cell volume and glucose uptake, cell telomerase activity and cellular senescence using senescence-associated-ß-galactosidase (SA-ß-gal) activity, and cell cycle phase by flow cytometry. Based on the present results, we have demonstrated that inhibition of cell growth in the human cancer cells can be induced by the cellular differentiation using high glucose metabolism with less cytotoxicity, rather than the chemical drugs with high cellular cytotoxicity for cancer chemotherapy treatment.

## Materials and methods

### Culture and treatment of cells

All cell culture media and chemicals were purchased from Sigma Chemical Company (USA) and Thermo Fisher Scientific (USA), respectively, unless otherwise specified. Human A-549 lung adenocarcinoma, mouse 3T3-L1 pre-adipocytes, and normal MRC-5 fetal lung fibroblasts were purchased from the American Type Culture Collection (USA). All types of cells were maintained in advanced-Dulbecco’s modified eagle medium (A-DMEM) supplemented with 3% fetal bovine serum (FBS) and 1.0% penicillin (10,000 IU/ ml)-streptomycin (10,000 μg/ml) at 37°C in a humidified atmosphere of 5% CO_2_. When cells were reached at 90% confluent status, cells were routinely sub-cultured by trypsin treatment. Other cells were treated with different PGZ concentrations.

### Analysis of cell viability by MTT assay

The cell viability and cytotoxicity were analyzed by 3-(4,5-dimethyl-2-thiazolyl)-2,5-diphenyl-2H-tetrazolium bromide (MTT) assay. Briefly, A549 cancer cells were grafted into a 6-well plate at the density of 2 × 10^4^ cells per well, and the cells were exposed in the A-DMEM media containing 0 (untreated control), 5, 10, 20, 50, 100, and 200 µM PGZ for 7 days. Afterward, cells were washed with Dulbecco’s phosphate-buffered saline (D-PBS) followed by treatment with 5 mg/ml MTT stock solution and incubation at 37°C. After 4 h, cells were washed with D-PBS. Formed formazan crystals were dissolved by DMSO treatment (15 min at RT) and transferred into a new 96 well-read plate, and absorbance intensity was measured with an ELISA microplate reader (Perkin Elmer, USA) at 570 nm wavelength. The viability rate of untreated controls was calculated as 100% compared to each treatment group of at least three replications. Further, the morphological alteration of treated cells was also observed under an inverted microscope (Nikon, Japan) equipped with a CCD camera.

### Analysis of cell proliferation and migration assay

After establishing a specific MTT concentration, a treatment concentration of 50 µM PGZ was decided to carry out the cell proliferation experiments utilizing population doubling time (PDT) assay. Untreated cells were taken as controls. Briefly, the A549 cancer cells were seeded in the A-DMEM media supplemented with 50 µM PGZ for up to 4 weeks. The media was replaced after every two days interval. Using a 0.25% trypsin EDTA solution, cells were harvested every week, and cell number changes were measured with a hematocytometer. The PDT was calculated as per the following formula: PDT = duration × log(2)/log (final concentration)−log(initial concentration). Further, a cell migration assay was performed using a wounded healing assay protocol. The untreated control and PGZ-treated A549 cells were seeded at the cell density of 1 × 10^6^ cells/well into a 6-well plate and cultured for 24 h until fully confluent. The straight scratches were made with a yellow pipette tip on the culture plate. After additional incubation for 48 h, the cell number in scratched one square (500 × 500 µm^2^) was counted under an inverted microscope (Nikon, Japan) equipped with a CCD camera.

### Analysis of cellular differentiation into adipocytes and cell volume

The adiposome-like droplets were frequently observed in nearly all of the A549 cancer cells at 1 week after 50 μM PGZ treatment. Neutral triglycerides and lipids in the adiposome-like vesicles were stained with Oil Red O solution. Briefly, the cells were washed in D-PBS and treated with a fixation solution overnight. And the cells were stained with 0.5% Oil Red O solution for 2 h. The lipid droplets stained with red color were investigated under an inverted microscope (Nikon, Japan). Further, the cell volume was calculated in the A549 cancer cells treated with 50 μM PGZ for 4 weeks. Every week, the cell diameter of over 100 cells was evaluated under an inverted microscope (Nikon, Japan) equipped with a CCD camera and image software (Nikon, Japan), followed by cell harvesting using 0.25% trypsin EDTA solution. The cell volume was calculated from cell diameter.

### Analysis of glucose uptake

The glucose uptake assay was quantified by measuring the medium’s glucose concentration after cell culture for up to 4 weeks. After every 1 week interval, culture media was collected, and the cell number was determined using a hematocytometer. The collected media’s glucose concentration was first measured with Accu-Chek blood glucometer (Roche, Germany) in over 20 replicates. The amount of glucose uptake was subtracted from the glucose amount present in the fresh A-DMEM and represented as consumed glucose concentration (ng/dl) per 1000 cells.

### Analysis of transcripts by reverse transcription-polymerase chain reaction (RT–PCR)

The expression level of glucose transporter 4 (GLUT4) and PPARγ transcripts was analyzed by reverse transcription-polymerase chain reaction (RT–PCR) assay. Briefly, the total RNA was purified with an RNeasy Micro Kit (GeneAll, Korea). After measuring RNA concentration, cDNA was synthesized with the help of an Omniscript RT kit (Qiagen, USA) with oligo-dT primers, and the reaction was carried out at 42°C for 1 h. After synthesizing cDNA, PCR was performed using a real-time PCR machine (Rotor-Gene Q, Qiagen, USA). The PCR amplification protocol was composed of denaturation for 15 s at 95°C, annealing for 10 s at 58–60°C, and extension for 16 s at 72°C in 30 cycles. And the PCR product was quantified by analyzing the threshold value (*C*_t_ value) with Rotor-Gene Q software. Glyceraldehyde 3-phosphate dehydrogenase (GAPDH) was used as a reference gene. Further, the amplified PCR products were harvested from each reaction tube in the real-time PCR machine, and the intensity was examined on the 1% agarose gel. The details of primers are previously described by Kim et al. (2018).

### Analysis of telomerase activity

The telomerase activity in 50 μM PGZ-treated A549 cancer cells was analyzed by a relative-quantitative telomerase repeat amplification protocol (RQ-TRAP) applying real-time PCR assay (Rotor-Gene Q, Qiagen, USA) and compared with those of 293T telomerase positive and normal MRC-5 fibroblasts. For protein extraction, harvested cells from each treatment were lysed with TRAPeze® 1X CHAPS cell lysis buffer (Cell Signal Tech., USA) at 4°C for 30 min, and centrifuged at 4°C for 20 min. RQ-TRAP assay using PCR machine (Rotor-Gene Q, Qiagen, USA) was detected with the 1 µg total protein of each sample, 0.02 µg of telomerase TS primer, and 0.04 µg of anchored return ACX primer in the 20 μl Rotor-Gene^TM^ 2× SYBR green mixture (Qiagen, USA). The TS and ACX primer sequence were previously described (Kim et al. 2017, 2018). Each reaction was first incubated for 30 min at 30°C and amplified in 40 cycles consisting of at 94°C for 30 sec and 60°C for 90 s. The threshold cycle values (*C*_t_) were determined with Rotor-Gene Q Series Software (Qiagen, USA) and the telomerase activity was relatively compared with 293T telomerase positive cells and MRC-5 fetal fibroblasts.

### Analysis of cell cycle by flow cytometry

After treatment with 50 µM PGZ for up to 4 weeks, the percentage of cell cycle phase was analyzed by flow cytometry (BD FACS Calibur, USA) according to previously published protocols (Jeon et al. 2015). Briefly, the cells were fixed with 70% ethanol for 30 min at 4°C. The cells’ nucleic acid (DNA) was stained with 50 µg/ml and propidium iodide (PI) for 30 min at 25°C. The cell cycle phase ratio was analyzed with Cell Quest Pro software (BD Bioscience, USA). The cell cycle phase was classified into G_1_, S, and G_2_/M as per each cell’s DNA content.

### Analysis of cellular senescence

After treatment with 50 µM PGZ for up to 4 weeks, the cellular damage and senescence were evaluated by SA β-gal activity using SA β-gal staining kit (Cell Signaling Technology, USA). Shortly after being fixed with a fixation solution, cells were stained with a β-galactosidase staining solution at 37°C overnight, followed by washing with D-PBS. Stained cells were examined under an inverted microscope (Nikon, Japan) equipped with a CCD camera and image program. The cells displayed with blue color were considered as positive for senescence.

### Statistical analysis

All data were presented as the mean and standard error of the mean (mean ± SEM). The significance of the statistical differences was analyzed by one-way analysis of variance (ANOVA) using SPSS statistics software (version 15.0, IBM, USA). Values were considered significant when *P* < .05.

## Results

### Cytotoxic determination of PGZ

**Figure 1.**
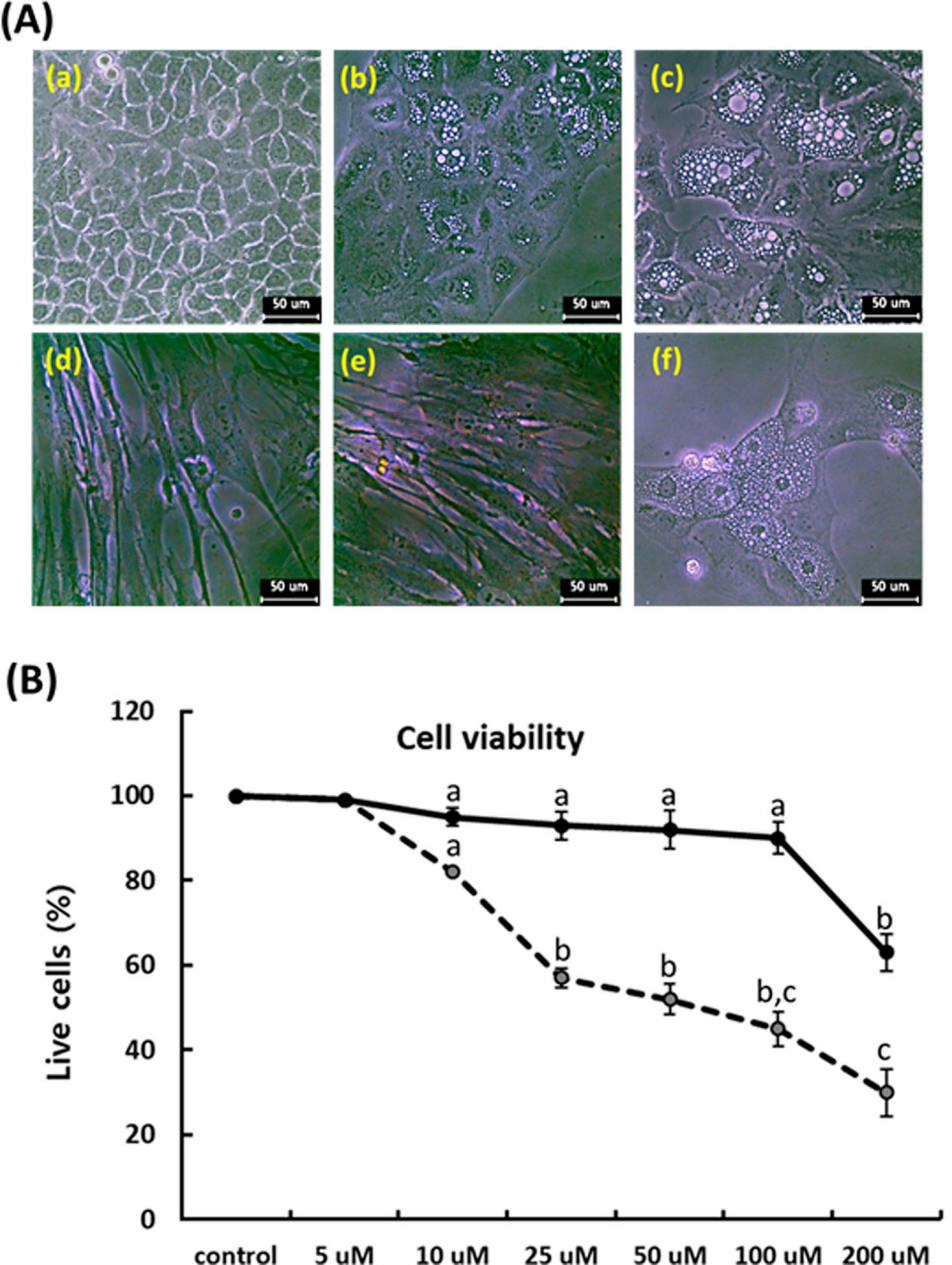
Analysis of morphological changes and cytotoxicity against PGZ by MTT assay in A-549 cancer (dotted line) and MRC-5 fibroblasts (solid line). A: Changes of cell morphology in untreated control A549 cancer cells (a), and 50 μM PGZ-treated A549 cancer cells for 1 week (b) and 3 weeks (c). Changes of cell morphology was not observed in untreated control (d) and 50 μM PGZ-treated MRC-5 fibroblasts (e) for 1 weeks. Morphological change with adiposome vesicles was also observed in 50 μM PGZ-treated 3T3-L1 pre-adipocytes (f) for 1 week. B: Inhibition curves of cell growth by MTT assay in A549 cancer cells and MRC-5 fibroblasts. a, b and c indicate different groups which are significantly different each other (*p *<* *.05, one-way ANOVA).

Following administration for 7 days in each PGZ concentration, the cellular cytotoxicity against PGZ was determined with cell proliferation assay using MTT solution in the A549 cancer cells, whereas MRC-5 fibroblasts were used as internal control cells. The adiposome-like vesicles were frequently observed at over 10 μM PGZ in the A549 (Figure 1), and the mean cell viability was respectively 98 ± 0.6, 82 ± 2.2, 57 ± 3.3, 52 ± 4.5, 45 ± 3.8, and 30 ± 4.6% in the 5, 10, 25, 50, 100 and 200 μM PGZ-treated A549 cancer cells, and the cell viability was significantly (*p* < .05) decreased at over 10 μM PGZ, compared with those of untreated control A549 cancer cells. Whereas the morphological alterations were not observed in the MRC-5 fibroblasts, and the mean survival rate was respectively 98 ± 0.3, 95 ± 0.6, 93 ± 2.3, 92 ± 3.7, 90 ± 4.1, and 63 ± 5.6% in the 5, 10, 25, 50, 100, and 200 μM PGZ-treated MRC-5 fibroblasts, compared with those of untreated control MRC-5 fibroblasts. The high cytotoxicity against PGZ was displayed at over 200 μM PGZ in MRC-5 fibroblasts.

### Analysis of population doubling time (PDT)

**Figure 2.**
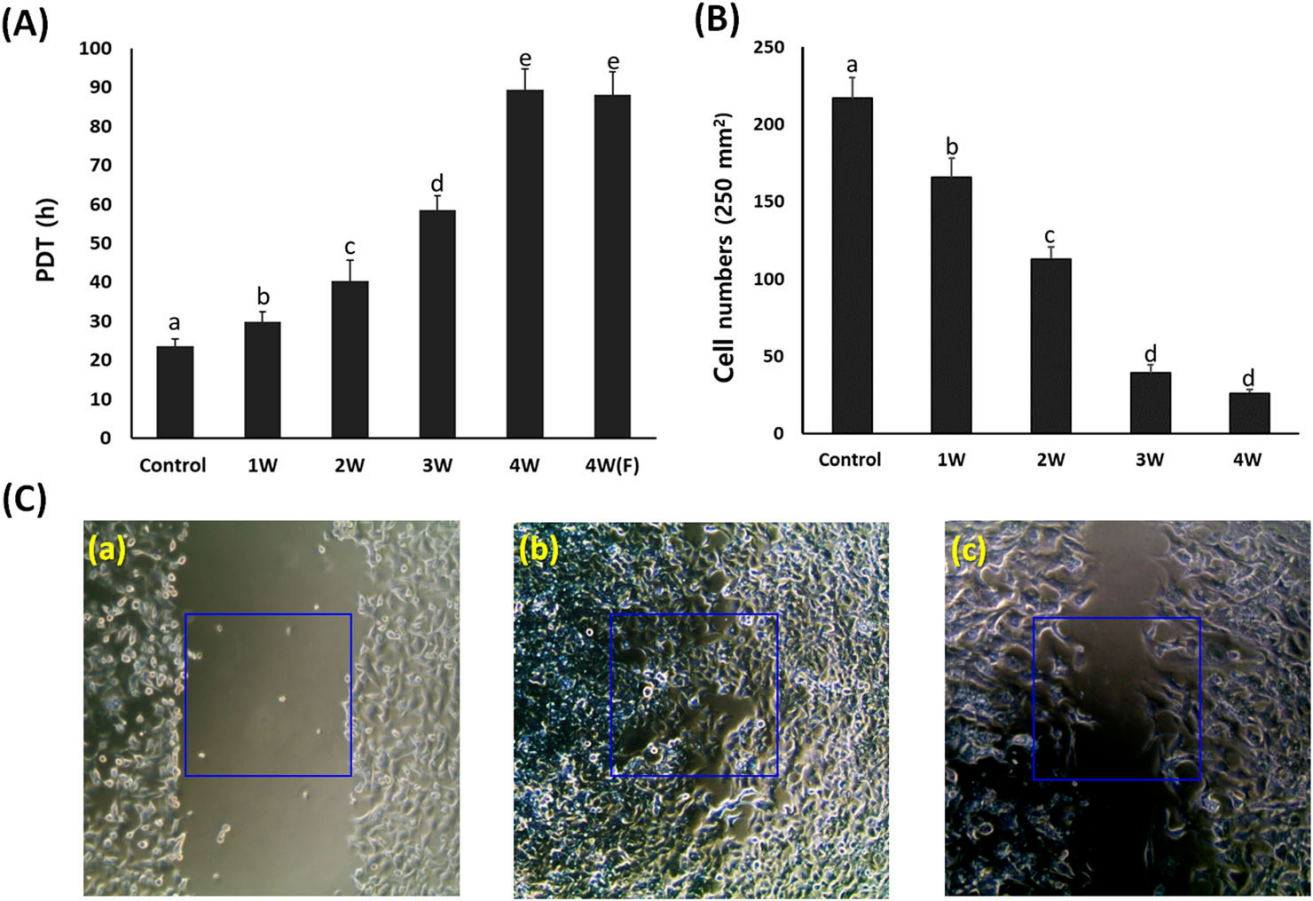
Analysis of cell growth by PDT (A) and wound healing assay (B and C) in A549 cancer cells treated with 50 μM PGZ. A: Weekly analysis of cell growth by PDT in A549 cancer cells treated with 50 μM PGZ up to 4 weeks. B: Cell number migrated into one square (250 μm^2^) in 50 μM PGZ-treated A549 cancer cells for up to 4 weeks C: Analysis of cell migration by wound healing assay in untreated control A549 cells after scratch (a), untreated control A549 cells at 48 h after scratch (b) and A549 cells treated with 50 μM PGZ for 4 weeks at 48 h after scratch (c). a, b, c, d and e indicate different groups which are significantly different each other (*p *<* *.05, one-way ANOVA).

A549 cancer cells cultured in the media containing 50 μM PGZ for up to 4 weeks were differentiated into adipocytes. The PDT was investigated by cell number (Figure 2). The mean PDT was 23.6 ± 1.89 in untreated control A549 cancer cells, whereas it was 29.8 ± 2.67, 40.4 ± 5.45, 58.5 ± 3.67, and 89.4 ± 5.33 h in the PGZ-treated A549 cancer cells. The PDT was significantly (*P* < .05) increased by the decrease of cell growth in proportion to days exposed to PGZ. Further, the A549 cancer cells exposed for up to 4 weeks in media containing 50 μM PGZ were further cultured for 1 week in the media containing 50 μM PGZ and fresh media without PGZ, and the PDT was 88.1 ± 5.87 h. After the administration of PGZ for 4 weeks, the cell growth was highly arrested, and the PDT was not significantly (*P* < .05) different between media containing 50 µM PGZ and fresh media without PGZ. Further, the cell migration capacity by wound healing assay was investigated in the treated A549 cancer cells. The cell number in the one square (500 × 500 μm^2^) was 217 ± 13.1 cells in untreated control cells, whereas it was found to be 165 ± 12.3, 113 ± 7.6, 39 ± 5.5, and 26 ± 4.6 cells in the one square of 50 μM PGZ-treated A549 cancer cells for 1, 2, 3 and 4 week(s). Migrated cell number was significantly (*P* < .05) decreased by the loss of cell migration and growth capacity in proportion to the increase of days exposed to PGZ.

### Analysis of cell volume

**Figure 3.**
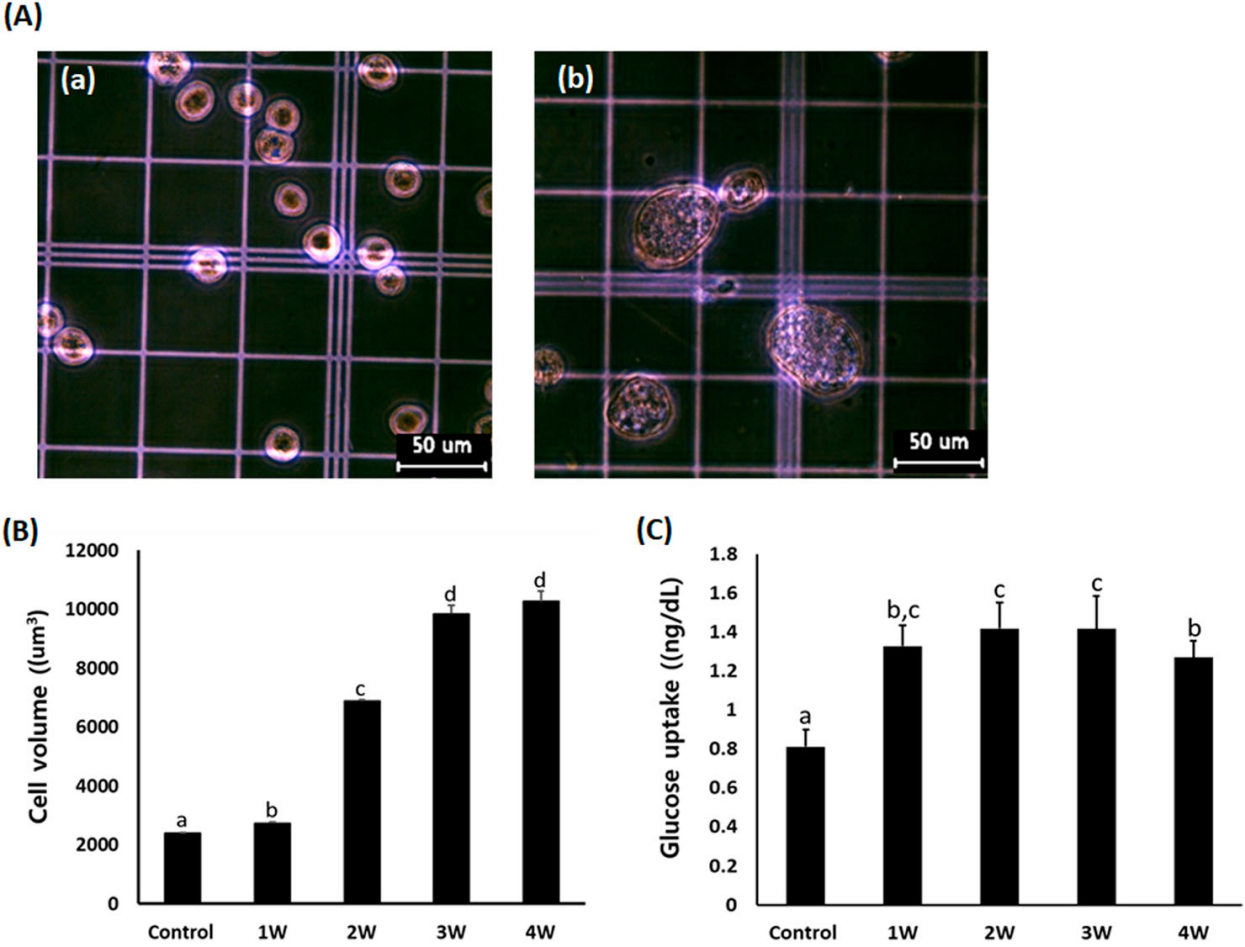
A: Analysis of cell volume in untreated control A549 cells (a) and A549 cells treated with 50 μM PGZ up to 4 weeks (b). B: Weekly analysis of cell volume in A549 cancer cells treated with 50 μM PGZ up to 4 weeks. C: Weekly analysis of glucose uptake in A549 cancer cells treated with 50 μM PGZ up to 4 weeks. a, b, c and d indicate different groups which are significantly different each other (*p *<* *.05, one-way ANOVA).

The increase in cell volume is considered as the main cellular property in the differentiating adipocytes. Based on the cell diameter (μm), cell volume was measured in the A549 cancer cells exposed in the media containing 50 μM PGZ for up to 4 weeks (Figure 3). The mean cell volume was 2385 ± 24.1 μm^3^ in PGZ untreated control A549 cancer cells, and it was 2734 ± 20.2, 6871 ± 57.2, 9843.6 ± 282.5, and 10267 ± 345.8 um^3^ in the media containing 50 uM PGZ for 1, 2, 3, and 4 week(s), respectively. The cell volume in the A549 cancer cells was significantly (*P* < .05) increased in proportion to the increasing PGZ exposure time. Further, the amount of glucose uptake was 0.81 ± 0.089 ng/dl in the untreated control A549 cells, and 1.32 ± 0.10, 1.41 ± 0.13, 1.42 ± 0.17, and 1.37 ± 0.09 ng/dl in the 50 μM PGZ-treated A549 cancer cells. The amount of glucose uptake was significantly (*P* < .05) increased in the A549 cells exposed to 50 μM PGZ.

### Analysis of cell differentiation into adipocytes

**Figure 4.**
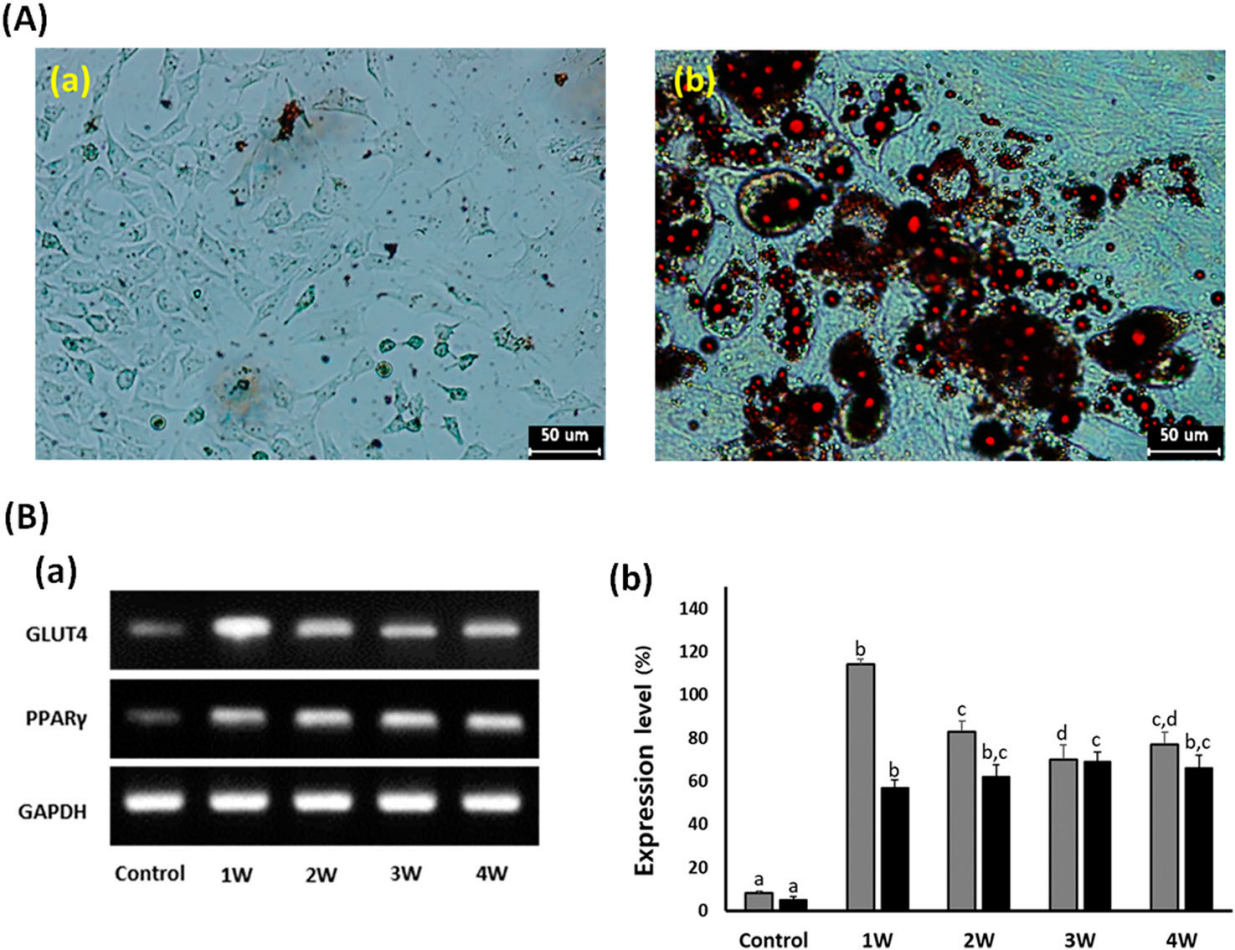
Analysis of cell differentiation into adipocytes in A549 cancer cells treated with 50 μM PGZ up to 4 weeks. A: Adiposomes were stained with oil red O solution in untreated control (a) and 50 μM PGZ-treated (b) A549 cancer cells. Accumulation of intracellular lipids in the cells were stained to red spots. B: Expression level of GLUT4 (▪) and PPARγ (▪) by RT-PCR in A549 cancer cells treated with 50 μM PGZ up to 4 weeks. a, b and c indicate different groups which are significantly different each other (*p *<* *.05, one-way ANOVA).

In the A549 cancer cells treated with 50 μM PGZ up to 4 weeks, adiposome-like vesicles were stained with Oil Red O solution (Figure 4(a)). The PGZ-treated A549 cancer cells were highly reacted to red color by the Oil Red O solution, implying that the A549 cancer cells have undergone cellular differentiation into adipocytes. Further, the expression level of GLUT4 and PPARγ was investigated by RT–PCR (Figure 4(b)). The relative expression level of GLUT4 and PPARγ transcripts was respectively 8 ± 0.91 and 5 ± 0.34%, compared with the GAPDH expression level. However, the expression level of GLUT4 and PPARγ was 114 ± 2.4 and 57 ± 3.6, 83 ± 4.9 and 62 ± 5.7, 70 ± 6.6 and 69 ± 4.4, and 77 ± 5.8 and 66 ± 6.1% in the 50 μM PGZ-treated A549 cells for 1, 2, 3, and 4 weeks, respectively. As exposure days to PGZ were increased, the expression level of GLUT4 and PPARγ was significantly (*P* < .05) increased in the differentiating A-549 cancer cells treated with 50 μM PGZ, compared with those of untreated control cells.

### Analysis of SA β-galactosidase and telomerase activity

**Figure 5.**
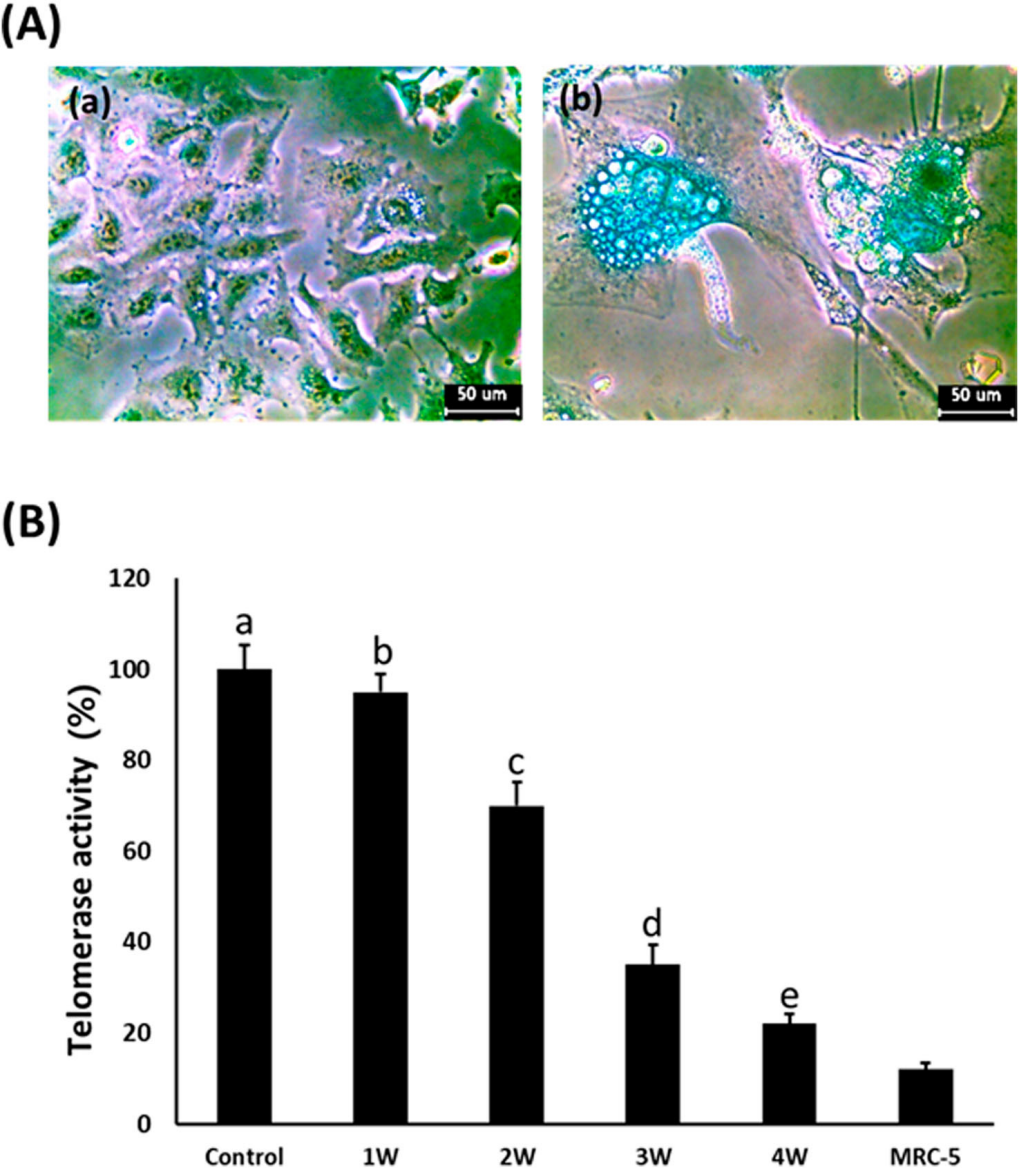
A: Analysis of senescence-associated-β-galactosidase activity in untreated control (a) and 50 μM PGZ-treated (b) A549 cancer cells up to weeks. After PGZ treatment, the morphological change of the cell to enlarged and flattened shape, and high activity of senescence-associated-β-galactosidase stained with blue color were displayed in A549 cancer cells treated with PGZ. B: Telomerase activity analyzed by RQ-TRAP in A549 cancer cells treated with 50 μM PGZ up to 4 weeks. a, b, c, d and e indicate different groups which are significantly different each other (*p *<* *.05, one-way ANOVA).

The cellular senescence and damage were estimated by senescence-associated β-galactosidase activity in the A549 cancer cells treated with 50 μM PGZ for up to 4 weeks (Figure 5(a)). After PGZ treatment, the morphological alternation of cells with a flattened shape as well as adiposome vesicles were commonly observed in the A-549 cancer cells treated with 50 μM PGZ, and the frequency and intensity of cells with high SA β-gal activity were also observed in the PGZ-treated A-549 cancer cells in proportion to the increase of days exposed to PGZ. Further, after treatment with 50 μM PGZ for up to 4 weeks, the relative telomerase activity was weekly measured in the A-549 cancer cells by RQ-TRAP assay, and the results (Figure 5(b)). The level of telomerase activity in the 50 μM PGZ-treated A549 cancer cells for 1, 2, 3, and 4 weeks was 95 ± 4.3, 70 ± 4.5, 35 ± 2.3, and 22 ± 1.5%, respectively, compared with those of untreated control cells. The telomerase activity level was significantly (*P* < .05) decreased in proportion to the increase of days exposed to PGZ and reached a level of normal MRC-5 fibroblasts.

### Analysis of phase of cell cycle

**Figure 6.**
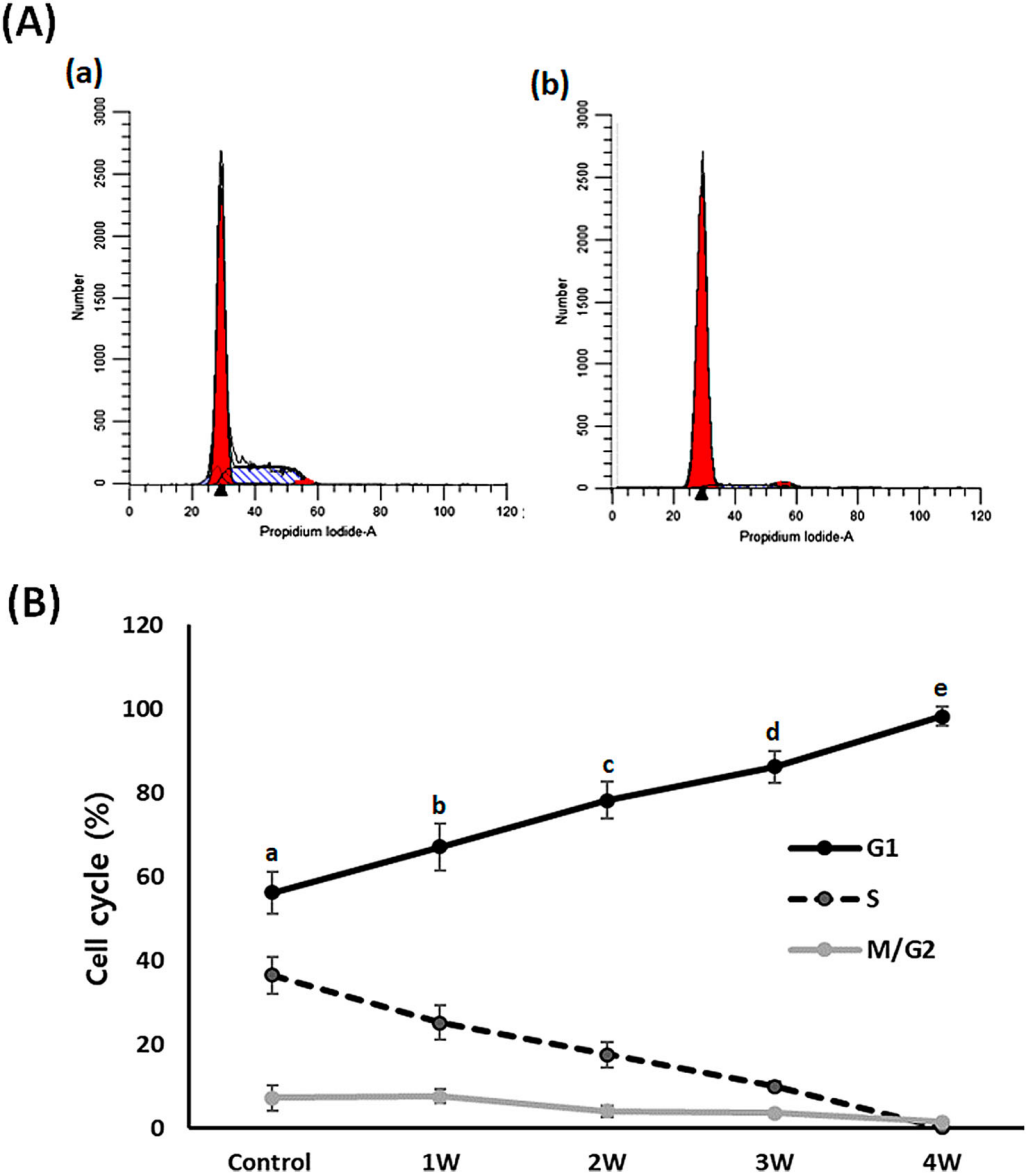
Analysis of cell cycle phase in A549 cancer cells treated with 50 μM PGZ up to 4 weeks. A: representative example in untreated control (a) and 50 μM PGZ-treated (b) A549 cancer cells for 4 weeks. B: The ratio of G1, S and M/G2 phase of cell cycles among each treatment week. a, b, c, d and e indicate different groups which are significantly different each other (*p *<* *.05, one-way ANOVA).

Flow cytometry assay was performed to evaluate the cell cycle events in the 50 μM treated A549 cancer cells (Figure 6). In the untreated control A-549 cancer cells, the proportions of cells in G1, S and G2/M phase were 56.2 ± 5.12, 36.5 ± 4.3 and 7.3 ± 2.98%, respectively. Whereas it was shown to be 67.1 ± 5.65, 25.2 ± 4.12 and 7.7 ± 1.54, 78.2 ± 4.42, 17.6 ± 3.01 and 4.2 ± 1.35, 86.2 ± 3.89, 10.1 ± 1.11 and 3.7 ± 0.34 and 98.2 ± 2.22, 0.2 ± 0.01 and 1.6 ± 0.13 in the A-549 cancer cells treated with PGZ for 1, 2, 3, and 4 weeks, respectively. The G1 phase of the cell cycle was significantly (*P* < .05) increased and the S phase of the cell cycle significantly (*P* < .05) decreased by administration of PGZ.

## Discussion

In the present study, cell growth inhibition via cellular differentiation into adipocytes was investigated in the A549 cancer cells that possess highly sensitive cellular characterization on adipogenesis progress as a model cell line. To induce adipogenesis, A549 cells were treated with PGZ, a kind of PPARγ activator, and their basic cancerous characterizations, such as cell growth, phase of the cell cycle, telomerase activity, and cell apoptosis were further investigated. It was demonstrated that PGZ treatment induces the cellular differentiation into adipocyte via morphological alteration and relevant gene expression, and loss of cancerous characterizations via arrest of cell growth, increase of G1 phase, down-regulated telomerase activity, and cellular apoptosis in the A549 cells. Whereas the cellular damage was weakly displayed in the normal cell lines, such as MRC-5 fibroblasts.

The PGZ is a kind of thiazolidinedione. The thiazolidinedione class chemicals, including rosiglitazone, troglitazone, and PGZ can improve glucose uptake and insulin sensitivity via oral administration and thus used as a common anti-hyperglycemic treatment for type 2 diabetes mellitus (DeFronzo et al. 2011). The PGZ effects on glucose metabolism, including glucose uptake and insulin sensitivity were induced by PPARγ activation. The adipogenesis is mainly controlled by high expression of C/EBPβ and C/EBPδ, and subsequently, PPARγ and C/EBPα, and the main function of PPARγ trigger adipocyte differentiation and lipid storage in predominantly adipose tissue (Kudoh et al. 2011; Lefterova et al. 2014). And the 3T3-L1 pre-adipocytes were differentiated into adipocytes under differentiation medium inducing high PPARγ activation (Lefterova et al. 2014). Previously we have shown that 3T3-L1 cells exposed to the culture medium containing DEX are easily induced to adipocyte-like cells with adiposome-like structures (Kim et al. 2018). In this study, lung A549 and breast MCF-7 adenocarcinoma cells were morphologically changed into adipocyte-like cells with adiposomes in the only DEX-containing culture medium and resulted in up-regulated GLUT4 and PPARγ expression (Kim et al. 2018). Similarly, other studies have demonstrated that A549 and MCF-7 cancer cells were highly sensitive and easily differentiated into adipocyte-like cells when exposed in DEX-containing medium, like 3T3-L1 cells (Buxant et al. 2015). Mesenchymal stem cells (MSC) derived from dental tissue displayed the cellular differentiation capacity into multi-lineage cell types; however, the cell differentiation into adipocyte and any distinguishing effects in the used MSC was not observed in the only DEX or PGZ-containing culture medium (data not shown). Further, PGZ treatment resulted in morphological alterations to adipocyte-like cells with adiposomes and high volume in the A549 cells. The adiposomes or lipid vesicles (droplets) containing lipid molecules are frequently found in the differentiating or maturing adipocytes (Jeon et al. 2015). The increased adiposomes containing neutral triglycerides and lipids were easily stained with Oil Red O staining solution (Jeon et al. 2015). In the present investigation, adiposome-like droplets were frequently observed in the A549 cells’ cytoplasm and easily stained with Oil Red O solution, implying that the PGZ-treated A549 cells have undergone cellular differentiation into adipocytes. Further, it has been exhibited that the differentiating and differentiated adipocytes are expanded by a drastic cytoskeletal remodeling, and therefore cell volume must be dramatically enlarged (Hansson et al. 2019). In accordance with these possibilities, A549 cells showed a dramatic increase in their cell volume under PGZ treatment and showed successful adipocyte differentiation. The present study has also demonstrated that PGZ treatment induces the upregulation of GLUT4 and PPARγ expression. Previously, GLUT4 and PPARγ transcripts are increased by PGZ treatment in the 3T3-L1 pre-adipocytes (Kudoh et al. 2011). Taken together, we thus assumed that PGZ treatment is unquestionably induced into adipocytes through activation of PPARγ in the A549 cells. Similarly, promoting differentiation into adipocytes was displayed by PGZ treatment in the 3T3-F442A cells, and it has suggested that PGZ might be used as a potential adipogenic agent for enhancement of glucose uptake and metabolism (Sandouk et al. 1993). However, none of the available previous studies on the cellular differentiation into adipocytes have demonstrated in human cancer cell lines. Thus, we have proposed the possibility of PGZ to be used as an effective, potent chemical for cancer chemotherapy via inducible differentiation into adipocytes. More interestingly and importantly, the arrest of cell cycle and growth was displayed in adipocyte-differentiated A549 cells after prolonged exposure to PGZ up to 4 weeks. It has previously been demonstrated that the adipocyte-like A549 and MCF-7 cancer cells are also exhibited to high inhibition of cell growth with down-regulation of telomerase activity after DEX treatment for 2 weeks (Kim et al. 2018). Generally, self-renewal and unlimited cell growth properties of both ESCs and iPSCs gradually disappeared when they undergo cellular differentiation to terminal cell types (He et al. 2009). Further, it has been reported that the G1 phase of the cell cycle in ESCs is even reduced or absent (Becker et al. 2007); however, the G1 phase of the cell cycle was gradually increased in the differentiated ESCs. The present results have shown that cell ratio at the G1 phase by the cell cycle delay was highly increased in the adipocyte-differentiated A549 cells treated with PGZ, compared with untreated control cells. In the previous studies, it has been pointed out that the G1 phase represents the most important step for cell fates, including cell proliferation, differentiation, senescence, and apoptosis (Blomen and Boonstra 2007). Moreover, the cells’ cycle phase reached cellular senescence or apoptosis is increased at the G0/G1 phase (Mao et al. 2012). Thus, we have agreed with previous studies that the G1 phase increase may be due to the consequence of cellular differentiation and/or cellular senescence and damage in the A549 cells treated with PGZ. Our results have shown that the telomerase activity is gradually decreased in the adipocyte-differentiated A549 cancer cells, along with increasing PGZ exposure days. An unlimited cell proliferation capacity without cellular senescence by most cancer cell lines is certainly related to the maintenance of telomeric repeats via high telomerase activity at the ends of the linear DNA of eukaryotes, including humans (Saretzki 2018). Whereas the down-regulation of telomerase activity in the cancer cells was induced to shorten or exhaust telomeric repeats and, subsequently, cellular apoptosis (Kim et al. 2017). We assumed that the down-regulation of telomerase activity in the differentiating adipocytes is induced to shorten telomeric repeats and inhibit cell growth and cellular apoptosis.

Moreover, the previous studies have considered that PPARγ activation with thiazolidinedione class chemicals, such as PGZ, can induce the cellular apoptosis pathway in the cancer cells as a kind of chemotherapeutic agent (Takahashi et al. 1999; Bodles et al. 2006; Elrod and Sun 2008), as shown in the present study. It has been demonstrated that tumor necrosis factor-alpha (TNF-α) secreted in early inflammatory reactions is a major pro-inflammatory cytokine, and TNF-α also induces the increased adiponectin expression as an adipokine via the PPARγ pathway (Zhou et al. 2015). It has been suggested that TNF-α induces the cellular apoptosis of human pre-adipocytes and adipocytes (Prins et al. 1997), implying that high TNF-α expression is probably related with the cellular apoptosis in the adipocytes. However, mechanisms and pathways behind PPARγ and PGZ-mediated cellular apoptosis is still unclear and not fully demonstrated in the present study. Basically, PPARγ is mainly regulated with lipid and numerous glucose metabolism and adipocyte differentiation; however, PPARγ is also related to the expression of more than 100 genes binding to DNA sequences as a transcription factor (Elrod and Sun 2008). The intrinsic mechanisms and pathways on growth inhibition induced by PGZ and adipocyte differentiation will further be investigated in the cancer cells. Further, high metabolic and energy activity constitutes another major concern regarding cancer cells. Most cancer cells display high metabolism capacity using aerobic glycolysis pathway to support their unlimited and high cell growth capacity, even in the presence of oxygen. And acidosis of the cells and microenvironment is induced by high glucose metabolism in the cancer cells (Gatenby and Gillies 2004). Even though most cancer cells exhibit acid resistance, the accumulation of hydrogen ion leads to the deleterious effect of the cell growth.

Morphological alterations with adiposomes and cellular cytotoxicity were induced by PGZ treatment in the different cancer cell lines, including MCF-7 and AGS cells (data not shown). More importantly, normal cells, such as MRC-5 fibroblasts, displayed extremely less cytotoxicity and sensitivity against PGZ treatment. Thus, the activation of the PPARγ gene demonstrated to be a key regulator for adipogenesis, and the cellular differentiation into adipocytes might evidently be considered as a chemical for effective chemotherapy or alternative or combined cancer treatment. In accordance with these observations, other types of safer and less cytotoxic PPARγ-targeting chemicals can be developed and used as potential chemotherapeutic agents.

Interestingly, MSCs have a peculiar feature differentiated into various types of mesenchyme cells, such as chondrocytes, osteocytes, and neurocytes (Jeon et al. 2015). Thus various types of cancer can also be investigated for their differentiation ability into other types of cell lineages (such as chondrocytes, osteocytes, and neurocytes), depending on the characterizations and origins of the cancer cells. Moreover, the effects of PGZ are to be carefully investigated in different types of cancer cell lines and in vivo treatment for prolonged dose time. In conclusion, the present study has shown that with the activation of PPARγ by PGZ, A549 cells can be successfully differentiated into adipocytes, showing all the required morphological alterations, adiposome vesicle formation, and high expression of several adipokines. Thus, this study has evidently demonstrated that differentiation therapy into adipocytes using high glucose metabolism of cancer cells might be effective in cancer treatment, rather than conventional chemotherapeutic chemicals targeting cellular cytotoxicity.
